# High-intensity interval exercise training for public health: a big HIT or shall we HIT it on the head?

**DOI:** 10.1186/s12966-015-0254-9

**Published:** 2015-07-18

**Authors:** Stuart J.H. Biddle, Alan M. Batterham

**Affiliations:** Institute of Sport, Exercise & Active Living, Victoria University, Footscray Park, 8001 Melbourne, VIC Australia; Health & Social Care Institute, Teesside University, Borough Road, TS1 3BA Middlesbrough, UK

**Keywords:** Exercise, Physical activity, Public health, Psychological affect, Enjoyment, Barriers, Correlates, Darwinian Medicine, Scalable, Straw man fallacy

## Abstract

**Background:**

The efficacy of high-intensity interval training for a broad spectrum of cardio-metabolic health outcomes is not in question. Rather, the effectiveness of this form of exercise is at stake. In this paper we debate the issues concerning the likely success or failure of high-intensity interval training interventions for population-level health promotion.

**Discussion:**

Biddle maintains that high-intensity interval training cannot be a viable public health strategy as it will not be adopted or maintained by many people. This conclusion is based on an analysis of perceptions of competence, the psychologically aversive nature of high-intensity exercise, the affective component of attitudes, the less conscious elements of motivated behaviour that reflect our likes and dislikes, and analysis using the RE-AIM framework. Batterham argues that this appraisal is based on a constrained and outmoded definition of high-intensity interval training and that truly practical and scalable protocols have been - and continue to be - developed. He contends that the purported displeasure associated with this type of exercise has been overstated. Biddle suggests that the way forward is to help the least active become more active rather than the already active to do more. Batterham claims that traditional physical activity promotion has been a spectacular failure. He proposes that, within an evolutionary health promotion framework, high-intensity interval training could be a successful population strategy for producing rapid physiological adaptations benefiting public health, independent of changes in total physical activity energy expenditure.

**Summary:**

Biddle recommends that we focus our attention elsewhere if we want population-level gains in physical activity impacting public health. His conclusion is based on his belief that high-intensity interval training interventions will have limited reach, effectiveness, and adoption, and poor implementation and maintenance. In contrast, Batterham maintains that there is genuine potential for scalable, enjoyable high-intensity interval exercise interventions to contribute substantially to addressing areas of public health priority, including prevention and treatment of Type 2 diabetes and cardiovascular disease.

In this paper, we debate the pros and cons on ‘high-intensity interval training’ (HIT) as a public health strategy. The paper is the product of a debate held at the ISBNPA Conference in Edinburgh in June 2015.

PRO: Alan M Batterham

## High-intensity exercise training could be a big HIT: Time to ditch the dogma of traditional physical activity promotion

### Background

High-intensity interval training (HIT) has been defined as either repeated short (<45 s) to long (2–4 min) bouts of rather high (not maximal) intensity exercise, or short (≤10 s, repeated-sprint sequences) or long (20–30 s, sprint interval session) all-out sprints, interspersed with recovery periods [1]. Since the idea of engaging in relatively low-volume HIT gained traction as a potentially viable means of conferring multiple health benefits [2], some prominent researchers have voiced their opposition [3]. The argument - well-rehearsed on the conference circuit and social media - goes something like this: “Of course HIT works, why wouldn’t it? But it won’t have any impact on public health if no one does it”. Such arguments propose that HIT has high efficacy but low effectiveness [4]; simply, that it *does* work, under optimal controlled circumstances with full compliance, but it *will not* work in practice. Herein, I challenge this position.

### Discussion

In his magnum opus, *On Liberty*, John Stuart Mill stated that “He who knows only his own side of the case knows little of that.” [5]. This philosophy informs my approach in this debate. I shall first debunk the main counterarguments to engaging in HIT, exposing the strategy and tactics used by opponents to discredit HIT as a valuable exercise option. Secondly, I shall seek to convince the reader that HIT could impact public health.

#### Typical criticism of HIT

Even for HIT detractors its efficacy is not in question^1^; clearly, engaging in HIT results in a broad spectrum of cardio-metabolic benefits [6–10]. Rather, the main critique of HIT is that not many people will be willing to engage (poor *reach*) and that in those who do participate there will be poor adherence and high attrition, such that effectiveness will be low. For example, Hardcastle *et al.* [11] argue that sprint interval training (SIT) is “inappropriate for a largely sedentary population”. The authors contend that SIT is likely to be perceived as too hard, leading to avoidance of adoption of the activity, and that those who do participate will drop out due to the associated negative feelings (affect). Here, the authors commit the logical fallacy of the straw man, presenting a particular form of HIT that is easy to knock down. Hardcastle *et al.* [11] are careful to use “Sprint Interval Training” in their title, delimiting the article to protocols involving repeated all-out 30-s bouts of cycle ergometer exercise (Wingate tests). This specific form of training is just one of many possible permutations in HIT programming within the definition given above [1]. Indeed, even ardent proponents of HIT concede that Wingate-based HIT is “extremely demanding and may not be safe, tolerable or appealing for some individuals” [7]. The field has moved on; many research groups are striving to develop and evaluate more practical HIT protocols, with potentially greater reach to a variety of clinical and apparently healthy populations [7, 12]. Hardcastle *et al.* [11] are clearly aware of this fact, as they cite the article from which the above quote is taken, but they have cleverly constructed a straw man to knock down. In short, no one is proposing Wingate-based SIT as a strategy to impact public health.

I have focused on the Hardcastle *et al.* article [11], as it provides an excellent recent example of the typical strategy and tactics adopted by the anti-HIT camp, involving cherry-picked evidence and straw man arguments. Readers will note that the title of their opinion piece – “Why sprint interval training is inappropriate for a largely sedentary population” – is a much more definitive sentiment than anything to be found in the article text, which is littered with expressions of uncertainty such as “*may* also be a barrier to”, “*may* lead to subsequent avoidance”, “*may* be inappropriate”, and “could” diminish intrinsic motivation and discourage adherence. This tactic is powerful, as many people will focus on the article title and the success of a straw man argument depends on the audience being uninformed or misinformed regarding the true position.

The research evidence typically advanced to justify attacks on HIT based on negative affect and lack of enjoyment is grounded in dual-mode theory [13, 14]. However, this body of work was not conducted using contemporary HIT protocols. One cannot take the liberty of extrapolating findings from continuous exercise above the ventilatory threshold to HIT protocols which are very different physiologically and motivationally. Based on this body of work, Biddle *et al.* claim that the ‘feel-good’ effect is unlikely during high-intensity exercise [3], a viewpoint repeated by Biddle from the podium at Sports Medicine Australia’s “Be Active” conference 2014. Recent research [15] counters this prevailing wisdom, with participants reporting comparable exercise enjoyment and confidence to engage in a HIT protocol vs. continuous moderate-intensity exercise and a preference for HIT over continuous moderate- or vigorous-intensity exercise.

The practice of ignoring evidence that contradicts their position, and repeating the anti-HIT mantra in publications, on social media, and at conferences, is an example of “blame gossip” in established-outsider relations [16]. Certainly, discourse is significant in the construction of ideologies and the attempted marginalisation of one group by another. In blame gossip, ‘evidence’ is discussed by the ‘established’ (here, HIT detractors) which is least flattering to an ‘outsider’ group (here, HIT proponents), attempting to create a favourable ‘we’ image and an unfavourable ‘they’ image [16].

### HIT could impact public health

I argue that HIT deserves a prominent place among a smörgåsbord of physical activity and exercise options [17]. A common misunderstanding among HIT detractors is the belief that the purpose of HIT interventions is to increase population physical activity levels. For example, in his ISBNPA conference abstract [18], Biddle speaks of “physical activity behaviour change”, and argues that “public health gains will be greatest if we help the least active become more active rather than the already active to do more.” Such statements completely miss the point of HIT interventions, where the emphasis is on using HIT as a throughput to achieve beneficial effects on myriad health outcomes. The key issue is that in exercise interventions (like HIT) the exercise behaviour is the independent variable, *not* the dependent variable. Health outcomes are what matter to public health commissioners and policy makers – not physical activity levels per se [19]. The distinction is critical because, as with efforts to prevent the increase in obesity and type 2 diabetes [20], we have spectacularly and persistently failed to promote physical activity [21].

One explanation proposed for the failure of physical activity promotion is the focus to date on health benefits, rather than acute and chronic psychological well-being [21]. However, Occam’s Razor teaches us to select the simplest, most elegant explanation of the available evidence amongst competing theories [22], and the answer could well lie in our biological heritage. Drawing on evolutionary biology and psychology, there is apparently no innate drive to be substantially physically active [23–26]. Rather, in the period in which the current human genome was selected physical activity energy expenditure was inextricably linked to – indeed, driven by – the procurement of food [27]. In the current environment, this link is broken resulting in the low physical activity energy expenditure typically observed. Supporting this assertion is the fact that countries with a low prevalence of insufficient physical activity are generally those whose economies rely on physical labour [21]. Therefore, for the majority of us in high-income industrialised countries, engaging in regular and frequent physical activity behaviours requires a conscious cognitive effort [25]. Consequently, the argument from detractors that HIT will not be effective because it requires high levels of motivation is redundant. From a Darwinian medicine perspective *any* sustained physical activity or exercise behaviour requires a high level of motivation, as there is no innate drive for it.

In the final section of my pro-HIT case, I advance the hypothesis that a public health campaign based on explaining the mismatch between our genes and our environment – and its consequences for chronic disease [28] – might be more effective than health promotion based on the carrot of psychological well-being. Consumer knowledge that in a Darwinian sense it is perfectly ‘natural’ to have low physical activity levels when there is no longer a need to hunt for and gather food - but that this abrogation comes with a price of chronic disease - could provide the requisite ‘cognitive push’ to counter the gene-environment mismatch. Although we might well require a certain (high) threshold of physical activity for normal physiologic gene expression and health [29] it is unlikely that as a population we will ever return to the high average physical activity levels of our Palaeolithic ancestors [23, 25]. It follows logically that time-efficient interventions like HIT [30, 31] – that do not necessarily require expensive specialist equipment or facilities [32, 33] - can help us fight chronic disease.

### Summary

There is great potential for HIT interventions to contribute to addressing areas of public health priority, including prevention and treatment of Type 2 diabetes and cardiovascular disease. As a potent weapon in waging war on non-communicable disease, HIT should be embraced.

#### CON: Stuart JH Biddle

## High-intensity exercise training (HIT) for public health: let’s HIT it on the head

Physical activity has become an important element of health promotion in many countries over the past few decades. Using the behavioural epidemiological framework [34], before we can roll out successful behaviour change solutions to address the pandemic of societal physical inactivity, we need to be able to identify a). the key health benefits and risks of different types of physical activity, b). the current levels of physical inactivity in the population, c). the correlates, determinants, and barriers of physical activity, and d). the types and ingredients of successful physical activity behaviour change interventions.

Regarding the health benefits of physical activity, it has been known for many years that a variety of dose-response curves exist [35]. Essentially, up to a point, higher doses of physical activity, such as intensity and duration, will yield additional physical health benefits. *This is not being disputed here*. But in order to determine if more physically demanding forms of physical activity are viable as a public health strategy, we need to consider the correlates and barriers to such behaviours, and whether the behaviours themselves will be adopted. It is largely pointless if some forms of physical activity are shown to produce significant health gains if few people adopt the behaviours.

### Defining ‘high intensity exercise’ (HIT)

In recent years, physiologists have shown a great deal of interest in the effects of very high intensities of physical activity. Often termed ‘high intensity interval training’ or ‘high intensity exercise training’, the catchy abbreviation of HITT, or simply ‘HIT’, has been adopted. Gibala and McGee [8] have defined HIT as “repeated sessions of relatively brief intermittent exercise, often performed with an ‘all-out’ effort or at an intensity close to that which elicits VO_2peak_ (*i.e.*, =/>90 % of VO_2peak_)” (p.58). These may last from a few seconds to several minutes, with periods of rest or low-intensity exercise. In referring to HIT, therefore, I am talking about very high exercise intensity, not just increasing intensity to the normal ‘vigorous’ range within the ubiquitous ‘moderate-to-vigorous’ (MVPA) nomenclature.

### Defining public health

The World Health Organisation (WHO) says that public health “refers to all organized measures … to prevent disease, promote health, and prolong life among the *population as a whole*. Its activities aim to provide conditions in which people can be healthy and *focus on entire populations, not on individual patients or diseases*” (http://www.who.int/trade/glossary/story076/en/) (emphasis added). Therefore, public health strategies, such as those aimed at tackling the pandemic of physical inactivity, need to attempt to reach the largest population possible. The greatest public health gains will be made by creating at least small changes but across large populations. This is in contrast to large gains in health but in only a small minority of people. The latter will leave the health of the population largely unaffected.

### Correlates of physical activity

To understand how best to promote more physical activity at the population level we need to understand what factors are associated with greater or lesser levels of physical activity (*i.e.*, correlates and barriers). These can be numerous and will sometimes differ by population and type of physical activity.

#### Perceptions of competence

Feelings of confidence and competence are key psychological drivers of participation. One facet of this is ‘self-efficacy’ which is a perception of one’s confidence to undertake a certain behaviour. This is an oft-cited construct underpinning behavioural choice and maintenance [3]. While undertaking short bouts of exercise may give people confidence that they can undertake the behaviour, the strenuous nature of HIT might undermine confidence, particularly regarding their ability to sustain such extreme behaviour over time. This is related to the nature of HIT being psychologically aversive, as discussed later.

#### Affective responses

The Dual-Mode Model of affective responses to exercise makes a number of propositions concerning the association between exercise intensity and affective (feeling) states [36, 37]. Proposition 4 states that “affective responses during strenuous exercise unify into a negative trend as the intensity of exercise approaches each individual’s functional limits” (p. 222) [36]. While a rebound to more positive feelings is predicted on completion of the exercise, I propose that such aversive psychological states *during exercise* will predict drop out or a marked reduction in exercise intensity over time during self-regulated exercise bouts. This is likely to be an explanation for results from a trial where inactive overweight adults were randomised to either an aerobic interval training group (AIT), maximal volitional intensity training (MVIT), or a walking group [32]. The walking group had fewer adverse events and adherence was particularly low in the two high intensity conditions. Given that this was a test of a ‘real world’ intervention, and yet adverse events and drop out were much higher in HIT conditions, one could argue that such an exercise protocol failed its test of public health applicability. Perhaps this is why army drill sergeants and some ill-informed teachers have, for many years, used vigorous exercise as a form of punishment?

One important factor often claimed to be associated with participation in physical activity is enjoyment, although the evidence is a little more complex than one might imagine [3]. On one occasion a former physiology colleague, when questioned about HIT being appropriate for all but a few people, stated that they enjoyed high intensity exercise more. When asked for supportive evidence, I was shown the paper by Bartlett *et al.* [38]. However, while ratings of enjoyment, using the Physical Activity Enjoyment Scale (PACES) [39], were indeed higher for the participants in a HIT running protocol than for continuous running, the sample comprised just 8 healthy, recreationally active men who were young, lean and fit. Such a study has no public health relevance. Moreover, the enjoyment ratings for the HIT group were taken after a 7-min cool down, not after 6x3 mins of high intensity interval running.

In looking at the PACES instrument in more detail, there are items signifying constructs where competent runners might be reporting their ‘liking’ for HIT through feelings of challenge and accomplishment. After all, it usually feels good after hitting one’s head against a brick wall! Although the paper doesn’t report individual item scores, I would not be surprised to see elevated scores, in this sample of competent runners, for items such as ‘it’s very gratifying’, ‘it’s very stimulating’, and ‘it gives me a strong sense of accomplishment’, particularly as all responses were made after the exercise bout. The dual-mode model would support such post-exercise feelings, but equally would predict feelings of considerable displeasure during exercise [36].

### Barriers

One of the most frequently mentioned barriers to taking part in physical activity is perceived lack of time [3]. For this reason, HIT has been seen as a positive initiative as it has the potential to reduce the time commitment to exercise. Notwithstanding that some have argued that the true time savings from HIT are quite small [11], it is important to understand that the stated barrier of lack of time may also reflect other psychological processes, such as values. By stating ‘I do not have time to exercise’ one is also providing a statement of the value attached to exercise. In short, it is not whether we have time, but how we chose to spend our time. To this extent, we all have time to meet national physical activity guidelines should we wish to.

The key issue, therefore, is not to find more time but to enable people to re-allocate their time to exercise because they value participation and its outcomes. To do this, psychological theory would suggest that behavioural intentions and behaviours will be enhanced by focussing on the positive affective element of attitudes more than instrument elements [40]. In other words, we need to boost people’s positive feelings about exercise. Making it harder and more painful is unlikely to do this.

### Behaviour change

Recent thinking on health behaviour change has highlighted the use of both conscious and less conscious processing for health behaviours [41, 42]. For example, the Behaviour Change Wheel [41] is based on behaviours being a function of capability, opportunity, and motivation, the latter comprising reflective and automatic processes. Reflective motivation is when people think through decisions and weigh-up pros and cons. I suggest for the large majority of people, the pros of HIT will be far outweighed by the cons. The Transtheoretical Model of behaviour change would support the view that when cons outweigh pros, the behaviour will not take place as the person will be stuck at the stage of contemplation [43].

Automatic processes of motivation will also be in operation for physical activity. This is where behaviours are triggered by much less conscious ‘gut feelings’, such as likes and dislikes. ‘Nudging’ people into health, by making environments more conducive and pleasant, seems most unlikely for HIT. HIT will require considerable psychological effort through planning and self-regulation and be accompanied by feelings of unpleasantness at a more sub-conscious level. In addition, it is logical to think that people’s capacity will be challenged with HIT, which is a similar argument made earlier about lacking perceived competence or confidence.

### HIT: time to ‘RE-AIM’ elsewhere?

The goal of the RE-AIM framework is to “encourage program planners, evaluators, … policy-makers to pay more attention to essential program elements including external validity that can improve the sustainable adoption and implementation of effective, generalizable, evidence-based interventions” (http://www.re-aim.hnfe.vt.edu/about_re-aim/index.html). RE-AIM stands for the five elements of Reach (the target population), Effectiveness or efficacy of the intervention, Adoption by targeted staff, settings, or institutions, Implementation consistency, costs and adaptations made during delivery, and Maintenance of intervention effects in individuals and settings over time. Although data to analyse HIT using the RE-AIM framework is not currently available, I speculate that reach will be very low, effectiveness will also be low at a population level (although could be effective for highly selected groups or settings), adoption will be low, implementation will be poor, and maintenance will be low. In short, the “external validity (for) sustainable adoption and implementation” of HIT is likely to be very poor indeed.

### Conclusion

There is currently no evidence supporting HIT as a viable public health strategy. Studies conducted to date are only efficacy studies, are limited by design (*e.g.*, lack of RCTs), have small sample sizes, have insufficient duration to determine the effects on longer term health outcomes, as well as adherence and changes on key psychological constructs known to be important for involvement in physical activity^2^. Moreover, even some of the main proponents of HIT have stated that “given the extreme nature of the exercise, it is doubtful that the general population could safely or practically adopt the model” [8] (p. 62). If this is the case, HIT cannot possibly be a viable public health strategy. Notwithstanding the failure of some other physical activity programs and interventions to create large or lasting effects, by making the behaviour harder to do certainly will not achieve the desired aim of having population-wide gains in physical activity and health. Instead of ‘more people, more active, more often’, I fear that HIT can only achieve having ‘few people, fitter, but not for long’.

### Rebuttal of Professor Biddle’s arguments

The live debate at ISBNPA 2015 hinged on two interrelated issues – how HIT is defined and whether it is associated with negative exercise affect (displeasure) likely to result in low adoption and poor adherence. First, let us re-examine the definition of HIT. Biddle states that he is referring to “very high exercise intensity, not just increasing intensity to the normal ‘vigorous’ range within the ubiquitous ‘moderate-to-vigorous’ (MVPA) nomenclature.” I, too, am talking about low-volume practical modes eliciting very high exercise intensity, above the ventilatory threshold in the ‘heavy’ intensity domain. However, it was clear from the live debate that Biddle is actually clinging to a much more constrained definition of HIT, as Sprint Interval Training (SIT) performed in 4-6 repeat Wingate tests involving 30 s of all-out maximal cycling against a high braking force on a specialized ergometer [8]. I reiterate that no one is arguing for implementing SIT as a public health approach.

Biddle’s denial that SIT has evolved into more practical, scalable HIT interventions - which result in similar cardio-metabolic responses to those observed with more extreme Wingate-based protocols - is illogical. In the live debate, Biddle derided my assertion that brisk incline walking [44, 32] is a viable form of HIT. He presumably based this criticism on the average intensity of brisk walking up a moderate incline being around 6 METs – the threshold for ‘vigorous’ intensity in MVPA nomenclature. Crucially, however, this logic ignores the influence of physical fitness - 6 METs is a high proportion of maximum aerobic power for many people. Indeed, in the Health Survey for England 2008 [45], overall 32 % of men and 60 % of women aged 16-74 were not physically fit enough to sustain walking at just 3 mph (1.34 m/s) up a 5 % incline, with this prevalence increasing with age. For a substantial proportion of the remainder, increasing speed and/ or grade would easily turn incline walking into a HIT modality. Biddle’s dismissal of incline walking as a HIT mode is remarkable, therefore, given his call to focus on the most inactive/ unfit segments of the population. The case for stair climbing as a scalable real-world HIT option for population health is also compelling. Emerging data from several groups – including Martin Gibala’s and ours – indicate that brisk intermittent stair climbing requires heavy-severe intensity exercise eliciting a substantial cardio-metabolic response.

Biddle’s seemingly irrational constraint of the definition of HIT is critical to his case, as defining HIT in its most extreme form creates a straw man that is easy to knock down. Without this definition, his case against HIT collapses. If my opponent will not acknowledge the evolution of SIT to HIT, then we shall have reached an impasse and he will be stuck in Plato’s Cave [46] as the field moves on.

The second main point of contention concerns the claim that HIT is associated with displeasure during the exercise leading to aversion and drop out. The ‘evidence’ used to sustain this assertion is derived from research on exercise affect within a dual-mode theory framework. The bottom line is that dual mode theory applies to continuous exercise above the ventilatory threshold, not to low-volume HIT with its built-in recovery periods. Anticipation of impending recovery, and the recovery period per se, results in more positive affect than continuous vigorous intensity exercise. For example, Jung *et al.* [15] reported that HIT intervals were conducted at a higher intensity than in the continuous vigorous intensity condition, yet affect was more positive in HIT, contrary to dual mode theory propositions. Compared to continuous vigorous exercise or longer-duration HIT, shorter HIT intervals (30 s) were associated with less perceptual drift in overweight, unfit participants [47]. Similarly, intervals of ≤60 s were found to be more enjoyable and to better maintain affect in overweight and insufficiently active adults [48]. In conjuring up negative consequences for HIT based on dual mode theory propositions, Biddle is hitting a screw with a hammer, to paraphrase Maslow [49]; dual mode theory simply cannot be applied to HIT.

Biddle argues that HIT can never be a viable population health strategy. In the live debate, he judged HIT interventions using the RE-AIM framework Self-Rating Quiz (http://bit.ly/1Ml69Nq), resulting in a rating of “Poor, needs serious attention”. Presenting this ‘result’ as a detached, objective finding is, of course, entirely specious.

In summary, practical, enjoyable and scalable HIT protocols exist that could form a viable population health strategy, implemented within an evolutionary health promotion framework. The case for hitting HIT on the head is dismissed.

### Rebuttal of Professor Batterham’s arguments

I thank Alan Batterham for taking part in this debate. William Penn, the English philosopher once said “In all debates, let truth be thy aim, not victory.”

Batterham argues that the field has moved on and no one is proposing Wingate-based sprint protocols for public health interventions. I am glad to hear that but this suggests that we need a better definition and delimitation of what ‘HIT’ really is. That’s a good outcome from this debate. However, I still feel my case is valid even with what Batterham argues are valid forms of exercise for public health within a ‘smörgåsbord’ of options. Wide choice is a good thing, but I argue that regardless of how many choices are available, few who need to exercise the most will choose the ‘hard’ option of HIT. Public health is about wide-reaching impact.

A second key issue raised by Batterham is that humans have no innate drive for physical activity and thus engagement requires cognitive effort. Psychologists now recognise that ‘motivation’ also includes ‘automatic’ processing. This could involve ‘nudge’ methods for behaviour change, where people are guided into behaviours with little or no conscious effort. This can be successful for low and moderate intensity physical activity, such as through environmental changes, but less likely, in my view, for high intensity.

Finally, I think we need to be careful about extrapolating findings on affective responses and ‘enjoyment’ from small studies to public health. For example, the study by Jung *et al.* [15] was on normal weight adults reporting some involvement in moderate, and a little vigorous, physical activity. The authors concluded that their study “highlights the utility of HIT in inactive individuals” (p.2), yet HIT showed progressively negative affective reactions compared to continuous moderate physical activity, in line with dual-mode theory [36].

I contend that HIT will never be taken up in sufficient numbers to have public health impact due to its aversive nature. Encouraging ‘more’ is good, but we should invest in promoting current physical activity guidelines and strive to assist the least active to become active (itself a challenge). I believe HIT will not achieve this.

### Joint conclusion

We are in agreement regarding the efficacy of HIT; clearly it produces rapid cardio-metabolic adaptations benefiting health. There is no other common ground between us, as we clearly disagree on the potential effectiveness of HIT and its likely impact on public health. Our polarized positions are informed by different conceptions regarding precisely what constitutes HIT, diverse interpretations of the literature on the relationship between exercise intensity and psychological affect, and contrasting philosophies for physical activity or exercise promotion. However, we are clearly both passionate about improving population health, and hope that our debate will stimulate further research to inform policy and practice. One key issue is to establish a commonly accepted definition of what constitutes ‘HIT’.

## Endnotes

^1^As with any intervention, a full evaluation of efficacy includes an assessment of potential harms, necessitating large long-term studies.

^2^SJHB is grateful to an anonymous reviewer for some excellent observations and suggestions noted here.

## Abbreviations

HIT: High-intensity interval training
RE-AIM framework: Reach, Effectiveness, Adoption, Implementation, Maintenance
